# Supplementary material for: Dynalign II: common secondary structure prediction for RNA homologs with domain insertions
Source: Nucleic Acids Res. 2014 Nov 21;42(22):13939–48. doi: 10.1093/nar/gku1172 (PMC4267632; doi:10.1093/nar/gku1172)
Supplement: SUPPLEMENTARY DATA [file supp_gku1172_nar-02021-z-2014-File012.zip › manual/Text/OligoWalk.html]

RNAstructure Command Line Help: OligoWalk


|  |  |  |
| --- | --- | --- |
|  | RNAstructure Command Line Help OligoWalk | - Contents - Index |
| OligoWalk is used to predict the accessible regions in an RNA target to oligonucleotide hybridization.  For usage information, please refer to the oligowalk/ directory in the RNAstructure repository.  This page will be updated soon... | | |
| Visit The Mathews Lab RNAstructure Page for updates and latest information. | | |
